# Prevalence of Positive Rapid Antigen Tests After 7-Day Isolation Following SARS-CoV-2 Infection in College Athletes During Omicron Variant Predominance

**DOI:** 10.1001/jamanetworkopen.2022.37149

**Published:** 2022-10-18

**Authors:** Jessica Tsao, Andrea Kussman, Nicole A. Segovia, Geoffrey D. Abrams, Alexandria B. Boehm, Calvin E. Hwang

**Affiliations:** 1 Department of Orthopaedic Surgery, Stanford University School of Medicine, Stanford, California; 2 Department of Civil & Environmental Engineering, Stanford University, Stanford, California

## Abstract

**Importance:**

The US Centers for Disease Control and Prevention shortened the recommended isolation period for SARS-CoV-2 infection from 10 days to 5 days in December 2021. It is unknown whether an individual with the infection may still have a positive result to a rapid antigen test and potentially be contagious at the end of this shortened isolation period.

**Objective:**

To estimate the proportion of individuals with SARS-CoV-2 infection whose rapid antigen test is still positive starting 7 days postdiagnosis.

**Design, Setting, and Participants:**

This case series analyzed student athletes at a National Collegiate Athletic Association Division I university campus who tested positive for SARS-CoV-2 between January 3 and May 6, 2022. Individuals underwent rapid antigen testing starting 7 days postdiagnosis to determine whether they could end their isolation period.

**Exposures:**

Rapid antigen testing 7 days after testing positive for SARS-CoV-2.

**Main Outcomes and Measures:**

Rapid antigen test results, symptom status, and SARS-CoV-2 variant identification via campus wastewater analysis.

**Results:**

A total of 264 student athletes (140 [53%] female; mean [SD] age, 20.1 [1.2] years; range, 18-25 years) representing 268 infections (177 [66%] symptomatic, 91 [34%] asymptomatic) were included in the study. Of the 248 infections in individuals who did a day 7 test, 67 (27%; 95% CI, 21%-33%) tests were still positive. Patients with symptomatic infections were significantly more likely to test positive on day 7 vs those who were asymptomatic (35%; 95% CI, 28%-43% vs 11%; 95% CI, 5%-18%; *P* < .001). Patients with the BA.2 variant were also significantly more likely to test positive on day 7 compared with those with the BA.1 variant (40%; 95% CI, 29%-51% vs 21%; 95% CI, 15%-27%; *P* = .007).

**Conclusions and Relevance:**

In this case series, rapid antigen tests remained positive in 27% of the individuals after 7 days of isolation, suggesting that the Centers for Disease Control and Prevention–recommended 5-day isolation period may be insufficient in preventing ongoing spread of disease. Further studies are needed to determine whether these findings are present in a more heterogeneous population and in subsequent variants.

## Key Points

**Question:**

Is the 5-day isolation period after SARS-CoV-2 infection that has been recommended by the US Centers for Disease Control and Prevention sufficient for infected individuals to receive negative test results?

**Findings:**

In this case series, 268 collegiate student athletes who tested positive for SARS-CoV-2 underwent rapid antigen testing starting 7 days after the initial positive test. At 7 days, the results of testing were still positive in 27% of the individuals tested, with a higher percent positive in symptomatic individuals and those infected with the Omicron BA.2 variant.

**Meaning:**

The findings of this study suggest that use of rapid antigen testing to aid in the decision to end isolation may be needed to prevent individuals with infection from leaving isolation prematurely.

## Teaser

This case series examines the results of rapid antigen tests in college student athletes with SARS-CoV-2 infection starting 7 days postdiagnosis.

## Introduction

One of the cornerstones of management during the COVID-19 pandemic has been to isolate individuals with infection to prevent viral spread. Guidelines for most of the pandemic have recommended 10 days of isolation.^1^ However, this prolonged period of isolation can lead to lost wages, workforce shortages, and other negative socioeconomic factors.^2,3^ In December 2021, the US Centers for Disease Control and Prevention decreased the recommended isolation period after a positive test from 10 to 5 days followed by 5 days of properly wearing a well-fitting mask. This decision was based on literature showing a transmission risk 2 to 3 days before and 8 days after symptom onset.^4,5^ However, these changes were made based on studies conducted during the development of the Omicron variant in 2022.

Rapid antigen test (RAT) positivity has been correlated with lower real-time polymerase chain reaction cycle threshold values, indicating higher viral loads and potential increased infectivity.^6,7,8,9^ Previous studies examining RAT results at day 5 have shown positivity rates higher than 40%.^10,11^ This percentage suggests that a substantial number of individuals may still be contagious when leaving isolation in the 5- to 10-day period. The purpose of this study was to estimate the rate of RAT positivity after 7 days of isolation in the context of the Omicron variants BA.1 and BA.2.

## Methods

In this case series, student athletes at a National Collegiate Athletic Association Division I school who tested positive for SARS-CoV-2 via polymerase chain reaction or antigen testing between January 3 and May 6, 2022, and were at least 2 weeks postcompletion of 2 doses of Moderna or Pfizer-BioNTech or 1 dose of Janssen vaccine against SARS-CoV-2 were eligible for inclusion in this study. Participants were placed in isolation housing for a minimum of 7 days from the date of their positive test, regardless of symptom onset date, and had the opportunity to test out (ie, exit) of isolation with a negative RAT starting on day 7. RATs were used per manufacturer instructions (Sofia SARS antigen FIA, QuidelOrtho, and Flowflex, Acon Laboratories Inc). Participants who tested positive could test again on days 8 and 9 or opt to a complete full 10-day isolation period without further testing. Participants self-reported symptoms occurring at any point during their isolation period on a post-COVID-19 clearance questionnaire that was filled out before exiting isolation and used to categorize each individual as symptomatic or asymptomatic. Herein we report the results of these test-out antigen tests. Campus wastewater data were used for circulating SARS-CoV-2 variant identification, using previously published methods (eMethods in the Supplement).^12,13,14^ This study followed the reporting guideline for case series.

This study was approved by the Stanford University Institutional Review Board. Because all data were deidentified and involved minimal risk, this study received a waiver of informed consent according to the US Department of Health and Human Services (45 CFR §46).

### Statistical Analysis

A multivariable Cox proportional hazards regression model was used to analyze the days until an individual who tested positive for SARS-CoV-2 receives a negative test result between variants and between participants who are symptomatic and asymptomatic. Multivariable logistic regression models were also used to estimate the odds of receiving a positive test on day 7 between variants and symptom status. All analyses were completed in RStudio, version 2021.09.1 (R Foundation for Statistical Analysis), using a 2-sided level of significance of *P* = .05.

## Results

A total of 264 student athletes (140 [53%] female, 124 [47%] male; mean [SD] age, 20.1 [1.2] years; range, 18-25 years) representing 268 infections (177 [66%] symptomatic, 91 [34%] asymptomatic) were included in the study. A total of 179 (67%) infections were detected via polymerase chain reaction testing; 89 (33%) infections were found via a RAT. A total of 183 (68%) infections occurred when the BA.1 variant was dominant and 85 (32%) with a dominant BA.2 variant.

**Figure 1.**
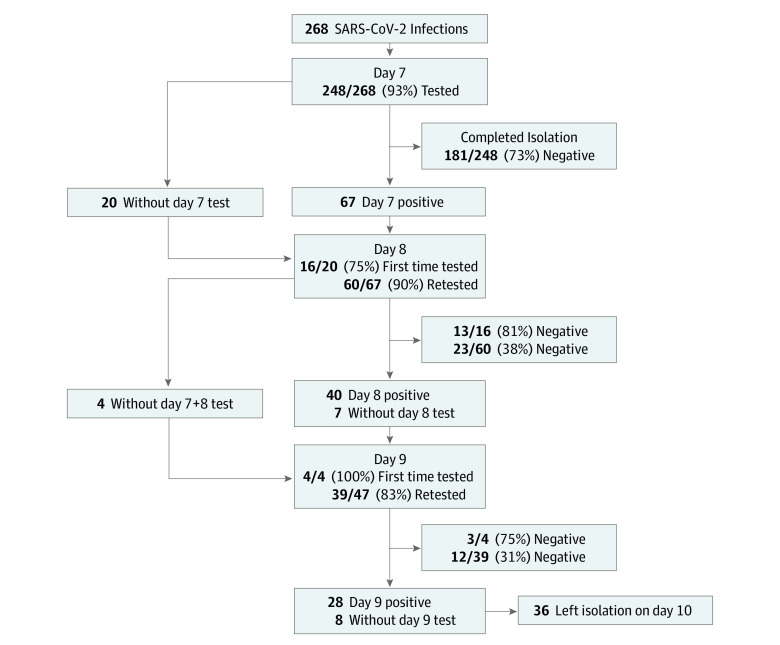
Flow Diagram of Antigen Test Results Test results of all 268 cases included in the cohort.

**Figure 2.**
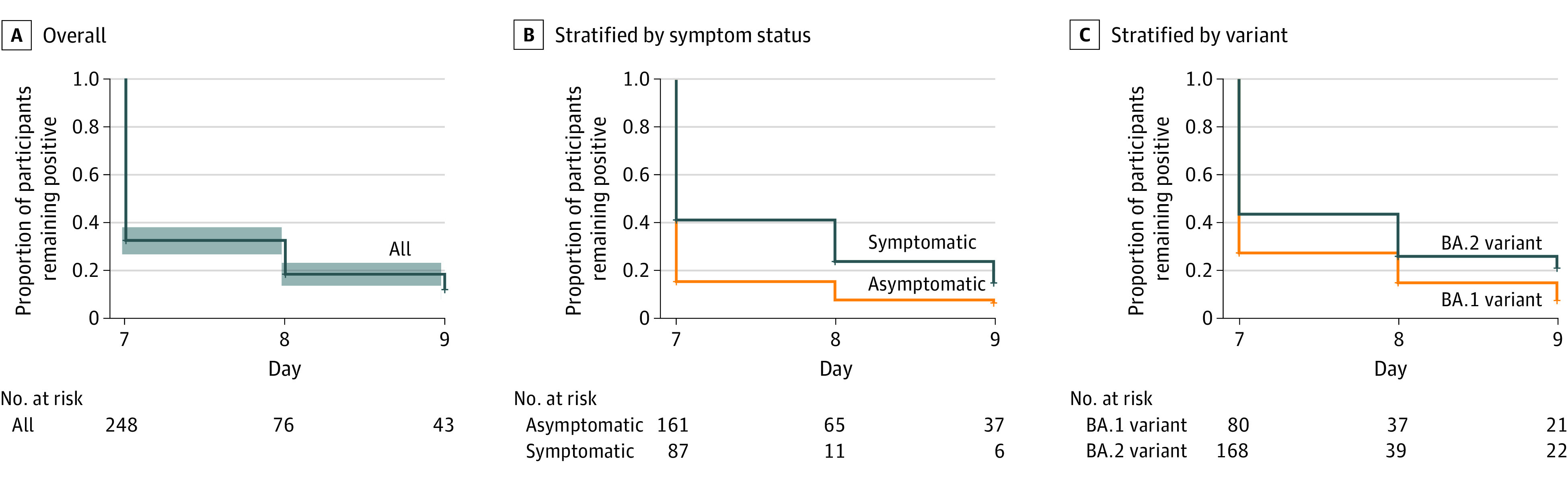
Proportion of Participants With Positive Results for SARS-CoV-2 After 7-Day Isolation Period The shaded area represents 95% CIs.

Of the 268 infections, 248 (93%) had a postpositive day 7 RAT performed, with 181 (73%) testing negative. On postpositive day 8, 76 tests were performed, with 36 (47%) testing negative, and on day 9, 43 tests were performed with 15 (35%) testing negative (Table 1). The remaining 28 student athletes who tested positive on day 9 were allowed to leave isolation on day 10 without further testing. A flow diagram of all the cases is shown in Figure 1. Individuals who tested positive on their first test were more likely to remain positive on subsequent tests with 62% of individuals testing positive on day 7 also testing positive on day 8, and 69% of those testing positive day 7 or 8 also testing positive on day 9. Kaplan-Meier curves showing the rate of persistent positivity based on symptomatic vs asymptomatic infections and the dominant strain are shown in Figure 2. Patients who were asymptomatic were significantly less likely to have a persistently positive RAT, with a hazard ratio of 0.60 (95% CI, 0.46-0.79; *P* < .001). Similarly, patients with the BA.1 variant were significantly less likely to have persistent positivity, with a hazard ratio of 0.69 (95% CI, 0.51-0.92; *P* = .01).

**Table 1.** Number of Individuals Testing Positive and Negative on Rapid Antigen Tests by Day From Initial Positive Test

Test day	No. (%)	
	Positive	Negative
Day 7	67 (27)	181 (73)
Day 8	40 (53)	36 (47)
Day 9	28 (65)	15 (35)

When looking only at day 7 tests, the positivity rate was significantly higher (57 of 161 [35%]) (Table 2) in individuals who had a symptomatic infection compared with those who were asymptomatic (10 of 87 [11%]), with an odds ratio of 3.92 (95% CI, 1.94-8.66; *P* < .001). Individuals who had the BA.2 variant were also more likely to have a positive initial test (32 of 80 [40%]), with an odds ratio of 2.29 (95% CI, 1.25-4.19; *P* = .007) compared with those infected by the BA.1 variant (35 of 168 [21%]) (Table 3).

**Table 2.** Number of Individuals Testing Positive and Negative on Their Initial Test Stratified by Presence of Symptoms by Day From Initial Positive Test

Test day	No. (%)			
	Symptomatic		Asymptomatic	
	Positive	Negative	Positive	Negative
Day 7	57 (35)	104 (65)	10 (11)	77 (89)
Day 8	3 (23)	10 (77)	0 (0)	3 (100)
Day 9	1 (33)	2 (67)	0 (0)	1 (100)

**Table 3.** Number of Individuals Testing Positive and Negative on Their Initial Test Stratified by Dominant Variant by Day From Initial Positive Test

Test day	No. (%)			
	BA.1		BA.2	
	Positive	Negative	Positive	Negative
Day 7	35 (21)	133 (79)	32 (40)	48 (60)
Day 8	1 (9)	10 (91)	2 (40)	3 (60)
Day 9	1 (25)	3 (75)	0	0

The dominant variant from January 3 to March 10, 2022, was BA.1; BA.2 became dominant from March 19 until the end of the study period (eFigure in the Supplement). There were no SARS-CoV-2–positive individuals between March 10 and March 19, 2022, when there was no clearly dominant strain.

## Discussion

In the cohort of National Collegiate Athletic Association Division I student athletes, 27% had a persistent positive RAT 7 days after their initial positive test. The rate of persistent positivity on RAT was even higher in participants with a symptomatic infection, with 34% testing positive. These rates are similar to those previously reported for the 5- to 10-day window, ranging from 17% to 58%.^10,15,16^

Individuals who tested positive on their first test were more likely to remain positive on subsequent tests with 62% of individuals testing positive on day 7 also testing positive on day 8, and 69% of those testing positive day 7 or 8 also testing positive on day 9 (Figure 1). The timing of antigen tests in this study was determined by the initial positive test rather than the onset of symptoms because university policy was to encourage individuals to test immediately on the onset of symptoms. As a result, symptoms typically did not precede a positive test by more than 1 day. Nevertheless, the symptomatic individuals in our cohort had a longer isolation period compared with other studies that used onset of symptoms.^10,17^ If day 0 had been symptom onset, it is likely that an even greater number of our cohort would have had persistent positive tests because they would have been eligible to test earlier.

There was also a significant difference in persistent positivity with the BA.2 Omicron variant independent of the presence of symptoms compared with the BA.1 variant. Together, these findings could call into question the current guidelines allowing for exit of isolation after 5 days without requiring additional testing to prevent further spread, particularly in the setting of newer variants, such as BA.4 and BA.5.

The use of testing before exiting isolation in the 5- to 10-day period to reduce the risk of persistent infectiousness is a common strategy used by universities, including Stanford University, and other organizations.^10,16,17^ Although the present study did not evaluate the association between RAT and infectivity, previous studies have shown a good correlation between RAT and viral load.^6,7,8^ One recent study found that a positive RAT on day 6 was only 50% predictive of a positive culture; however, a negative RAT on day 6 was 100% predictive of a negative culture.^17^ These results suggest the potential value of RAT to inform isolation duration, even beyond the previously recommended 10-day period or shorter than the 5-day period.

### Limitations

This study has limitations. Wastewater samples were used to infer the circulating variant rather than clinical samples because a large number of students in the cohort never underwent polymerase chain reaction testing, limiting the availability of samples for sequencing. However, wastewater data are specific to the university population and are likely to represent the variants in circulation well.^12,18,19^ The eFigure in the Supplement illustrates the overlap between case incidence and wastewater virus levels. Wastewater data were used as a surrogate for variant typing, but the efficacy of this approach with subsequent strains will need to be revisited.

Another limitation of this study is that the university discontinued mandatory surveillance testing partway through the study period; thus, there are fewer asymptomatic infections in the BA.2 variant cohort, which represents the latter half of the testing period. The use of multivariate and Cox proportional hazards regression analyses helps to minimize this potential confounder.

All participants in this study were college-aged, fully vaccinated, and had received booster doses if eligible, limiting the generalizability to unvaccinated or partially vaccinated populations and to the general population. We were also unable to assess whether individuals had any persistent symptoms at the time of their RAT, which has previously been associated with a positive RAT.^6^ Because testing out of isolation before 10 days was voluntary, not all individuals tested every day starting at day 7 and not all chose to test on their first eligible day. However, most participants (248 of 268) tested on day 7.

## Conclusions

More than a quarter of individuals in this case series had a positive RAT 7 to 10 days after their initial positive test, with even higher percentages of persistent positivity in individuals with symptomatic infections and the newer BA.2 variant. This finding suggests that a substantial number of individuals may still be contagious after completing the Centers for Disease Control and Prevention–recommended 5-day isolation period.

## Notes

Supplement. **eMethods.** Wastewater Analysis
eReferences
**eFigure.** Wastewater Variant Levels and Case Data
