# Supplementary material for: Prevalence of Positive Rapid Antigen Tests After 7-Day Isolation Following SARS-CoV-2 Infection in College Athletes During Omicron Variant Predominance
Source: JAMA Netw Open. 2022 Oct 18;5(10):e2237149. doi: 10.1001/jamanetworkopen.2022.37149 (PMC9579911; doi:10.1001/jamanetworkopen.2022.37149)
Supplement: Supplement. — eMethods. Wastewater Analysis eReferences eFigure. Wastewater Variant Levels and Case Data [file jamanetwopen-e2237149-s001.pdf]

Supplementary Online Content

Tsao J, Kussman A, Segovia NA, Abrams GD, Boehm AB, Hwang CE. Prevalence of positive rapid antigen tests after 7-day isolation following SARS-CoV-2 infection in college athletes during Omicron variant predominance. *JAMA Netw Open*. 2022;5(10):e2237149. doi:10.1001/jamanetworkopen.2022.37149

eMethods. Wastewater Analysis
eReferences
eFigure. Wastewater Variant Levels and Case Data

This supplementary material has been provided by the authors to give readers additional information about their work.

© 2022 Tsao J et al. *JAMA Network Open.*

eMethods. Wastewater Analysis

Samples from Stanford University were collected from a manhole that accesses a large sewer main that conveys wastewater from nearly 200 buildings on campus including student and faculty housing for 10,000 people. During the study period, 24-hour composite samples were collected 6 days per week. A temperature controlled autosampler was set at 4°C and collected a raw wastewater sample every 30 minutes; the sample was then placed into an Imhoff cone to settle the solids; the solids were collected and stored at 4°C before processing on the same day. Samples collected between 1 Jan 2022 and 6 May 2022 are included in this study. The following SARS-CoV-2 gene targets were measured in the samples: a pan-SARS-CoV-2 RNA target located in the nucleocapsid (N) gene, a mutation characteristic of Omicron BA.1 (del143-145), and a mutation characteristic of BA.2 (LPPA24S). The concentration of pepper mild mottled virus (PMMoV) was also measured as an endogenous RNA extraction control. The complete methods used to measure these targets including pre-analytical and analytical methods, and quality control and assurance procedures have been published in detail elsewhere[1-3] and are available as open access methods on protocols.io. Results are displayed as time series of N, del143-145, LPPA24S normalized by PMMoV, as we have done and justified previously[2]. Results are similar when plotted un-normalized by PMMoV (data not shown). Wastewater data are publicly accessible through the Stanford Digital Repository (https://doi.org/10.25740/kc480wq1182).

© 2022 Tsao J et al. *JAMA Network Open.*

eReferences.

1. Boehm AB, Hughes B, Wolfe MK, White BJ, Duong D, Chan-Herur V. Regional replacement of SARS-CoV-2 variant Omicron BA.1 with BA.2 as observed through wastewater surveillance. *Environ Sci Technol Lett*. 2022;9(6):575-580.
2. Wolfe MK, Topol A, Knudson A, et al. High-Frequency, High-Throughput Quantification of SARS-CoV-2 RNA in Wastewater Settled Solids at Eight Publicly Owned Treatment Works in Northern California Shows Strong Association with COVID-19 Incidence. *mSystems*. 2021;6(5):e0082921.
3. Wolfe M, Hughes B, Duong D, et al. Detection of SARS-CoV-2 Variants Mu, Beta, Gamma, Lambda, Delta, Alpha, and Omicron in Wastewater Settled Solids Using Mutation-Specific Assays Is Associated with Regional Detection of Variants in Clinical Samples. *Appl Environ Microbiol*. 2022;88(8):e0004522.

© 2022 Tsao J et al. *JAMA Network Open.*

eFigure. Wastewater Variant Levels and Case Data.

Wastewater data indicating levels of BA.1 and BA.2 with incident cases from student-athlete cohort. Incident cases is a 5-d centered running average of daily case data. Wastewater data shows a 5-d centered trimmed average of measurements of the N gene (present in variants), the del143-145 mutation (present in BA.1), and the LPPA24S mutation (present in BA.2) normalized by concentrations of PMMoV.

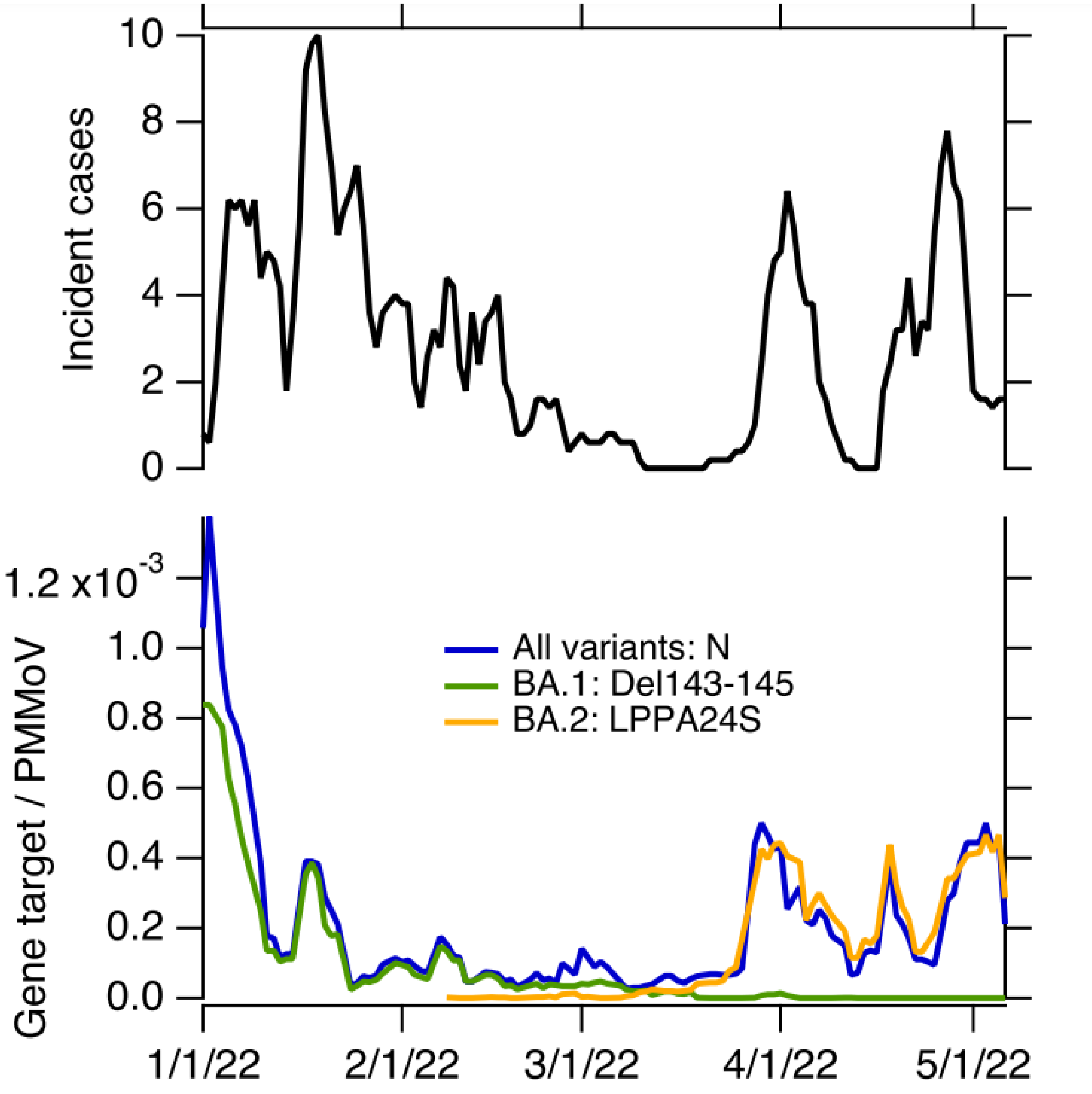

© 2022 Tsao J et al. *JAMA Network Open.*
